# From hypoxic pockets to daily routines: linking brain oxygenation and cognitive resilience

**DOI:** 10.3389/fnagi.2025.1534198

**Published:** 2025-01-29

**Authors:** Dian Jiao

**Affiliations:** Institute of Psychiatry, Psychology and Neuroscience, King’s College London, London, United Kingdom

**Keywords:** cerebral oxygenation, cognitive health, physical activity, social engagement, cognitive stimulation, neurovascular coupling, angiogenesis, neuroplasticity

## Abstract

The discovery of hypoxic pockets within the cortical regions has transformed the understanding of cerebral oxygen dynamics, revealing their dual role as both contributors to neuronal adaptation and potential precursors to dysfunction. These transient oxygen-deprived microenvironments play a pivotal role in neurovascular coupling, synaptic plasticity, and angiogenesis, processes crucial for maintaining cognitive resilience and neuronal health. Investigating hypoxic pockets within cortical regions is particularly relevant in aging populations and individuals with neurodegenerative conditions. Concurrently, research underscores the ability of physical, social, and cognitive activities to modulate brain oxygenation, offering natural, accessible interventions to optimize oxygen delivery and utilization. This study synthesizes findings from neuroimaging, behavioral science, and longitudinal studies, illustrating how daily routines can mitigate hypoxia-induced cognitive decline and promote resilience. By integrating insights from centenarians, hypoxia-adapted species, and multimodal intervention studies, this framework highlights the transformative potential of lifestyle-based strategies in addressing cerebral oxygen deficits. The findings advocate for an interdisciplinary approach to develop targeted interventions for public health, rehabilitation, and personalized cognitive care.

## Introduction

Cerebral oxygenation is the cornerstone of neuronal function and underpins cognitive health and resilience. The brain’s high-energy demands rely heavily on aerobic metabolism, which depends on a consistent and efficient supply of oxygen. Historically, the brain has been thought to receive oxygen uniformly through its dense vascular network, maintaining stable oxygenation across its regions. However, studies on vascular cognitive impairment and dementia (VCID) have highlighted the complex interplay between vascular health, metabolic processes, and cognitive function, demonstrating how disruptions in cerebral blood flow (CBF) and associated metabolic deficits contribute to cognitive decline (Popa-Wagner et al., 2014; Popa-Wagner et al., 2015). These foundational insights underscore the importance of neurovascular mechanisms in shaping oxygen dynamics across brain regions. Nevertheless, recent advancements in imaging technologies have disrupted this simplistic view, revealing the existence of localized hypoxic pockets — transient areas of oxygen depletion within cortical regions (Beinlich et al., 2024). These pockets, characterized by disruptions in capillary perfusion and oxygen delivery, create dynamic microenvironments where metabolic demand temporarily exceeds oxygen supply. For instance, microvascular occlusions can lead to hypoperfusion downstream, forming hypoxic regions distant from the primary blockage (Xue et al., 2022; Qiao et al., 2002), further underscoring that these deficits can arise even away from the initial site of vascular compromise. More importantly, these microenvironments play a dual role, being essential for physiological processes like neurovascular coupling, synaptic plasticity, and angiogenesis, while also increasing susceptibility to neuronal dysfunction when unresolved (Zhou et al., 2024; Zhang et al., 2024). These findings emphasize the importance of strategies aimed at mitigating the adverse effects of unresolved hypoxic states while leveraging their potential adaptive benefits.

Simultaneously, emerging evidence has highlighted the capacity of routine physical, social, and cognitive activities to modulate brain oxygenation, support neural health and cognitive outcomes (Adjetey et al., 2023; Hayes et al., 2014; Liu et al., 2023; Hakun et al., 2024). Those findings suggest the intriguing possibility that daily behaviors may serve as natural interventions targeting hypoxic regions and optimizing oxygen delivery to critical brain areas.

This paper explores the intersection of these discoveries, proposing that diverse, everyday activities not only improve regional brain oxygenation but also offer a practical and accessible strategy for maintaining cognitive health across the lifespan. By integrating insights from neurovascular physiology and behavioral science, this framework underscores the transformative potential of lifestyle interventions to address localized oxygen deficits, particularly in aging and neurodegenerative populations. To deepen this understanding, the discussion now turns to the mechanisms driving these effects, their implications for clinical practice, and future directions that could propel this promising field forward.

## Current understanding of brain oxygenation

The discovery of hypoxic pockets within the cerebral cortex has revolutionized our understanding of brain oxygen dynamics, challenging the long-held belief that the brain’s dense vascular network uniformly supplies sufficient oxygen to meet metabolic demands. In light of this discovery, advanced imaging techniques have uncovered transient, localized areas of oxygen deprivation, measuring 20–200 μm in diameter and lasting mere milliseconds to seconds (Beinlich et al., 2024; Bon et al., 2023). These pockets emerge when oxygen delivery falls short of metabolic needs, particularly during intense neural activity or when capillary perfusion is disrupted.

In healthy brains, these events occur sporadically and often activate adaptive responses such as angiogenesis or neurovascular remodeling, which enhance oxygen supply over time (Acker and Acker, 2004; Zhou et al., 2024). Yet, in pathological states, such as cortical spreading depression (CSD), traumatic brain injury, or neurodegenerative disorders, these mechanisms are often overwhelmed. Repeated or prolonged hypoxia in these contexts can impose a significant burden on brain tissue, driven by impaired neurovascular coupling and diminished vascular responsiveness (Takano et al., 2007; Veenith et al., 2016). While angiogenesis can partially counterbalance these effects, the severity and duration of hypoxia are shaped by the interplay between local metabolic demands and systemic influences (Acker and Acker, 2004).

Given their critical impact on brain function, hypoxic pockets have drawn significant attention in regions with high metabolic demand, such as the prefrontal cortex, which supports executive functioning, and the hippocampus, essential for memory formation (Murayama et al., 2017). Their spatial clustering and persistence highlight their pivotal role in influencing cognitive resilience and neural health. Emerging evidence from high-resolution imaging reveals that these hypoxic episodes often align with task-driven neural activity or arise in regions compromised by vascular dysfunction (Hoffmann et al., 2016; Stoica et al., 2022). Although each episode is brief, their cumulative effects—particularly under sustained stress—can persist for minutes to hours and progressively impair neural function if left unresolved (Friedrich et al., 2013; Takano et al., 2007). However, determining the precise frequency and overall burden of these episodes remains a pressing research challenge.

Adding to this complexity is the variability in oxygenation patterns across brain regions. For instance, the prefrontal cortex experiences notable oxygen fluctuations during cognitive tasks, whereas the occipital cortex—typically less susceptible—may become vulnerable under external stressors, such as hypobaric environments at high altitudes (Murayama et al., 2017; Hohenauer et al., 2022). These findings underscore the intricate balance between oxygen supply and demand, shaped not only by localized activity but also by external environmental conditions.

Despite significant advances in neuroimaging technologies, such as functional near-infrared spectroscopy (fNIRS) and high-resolution MRI, our ability to capture the fleeting nature of hypoxic events in real time remains limited (Herold et al., 2018; Salzman et al., 2022). The need for developing portable, high-resolution imaging technologies to directly link oxygen deficits to specific cognitive outcomes is imminent. Bridging these gaps in knowledge could pave the way for targeted interventions to reduce the occurrence of hypoxic pockets and protect cognitive health, particularly among older adults and those at heightened risk for neurodegenerative diseases.

### The activity-oxygenation connection

Daily activities exert profound effects on brain oxygenation through interconnected pathways, which enhance neuronal health and cognitive resilience. These activities, such as physical exercise, social engagement, and cognitive stimulation, play a critical role in modulating oxygen delivery and utilization. By targeting hypoxic pockets, they promote neurovascular adaptation, synaptic plasticity, and metabolic efficiency. Additionally, they facilitate angiogenesis, which helps resolve localized oxygen deficits, ensuring better oxygen distribution and utilization throughout the brain.

#### Physical activity effect

Physical exercise is a powerful modulator of cerebral oxygenation, inducing both immediate and long-term neurovascular benefits. Aerobic activities such as walking or running acutely increase cardiac output and cerebral blood flow (CBF) to meet heightened metabolic demands, effectively resolving transient oxygen deficits in hypoxic pockets by enhancing neurovascular coupling (Willie et al., 2011; Mulser and Moreau, 2023; Beinlich et al., 2024). Exercise-induced increases in nitric oxide production improve vasodilation and capillary perfusion, contributing to better oxygen delivery in under-oxygenated brain regions such as the hippocampus and prefrontal cortex (Arefirad et al., 2022; Beinlich et al., 2024). Advanced imaging techniques further reveal regional specificity in these effects: motor tasks requiring balance and coordination primarily activate the cerebellum and motor cortices, whereas aerobic exercises enhance functional connectivity in prefrontal and temporal areas vulnerable to cognitive decline (Benarroch, 2022; Li et al., 2016; Hayes et al., 2014).

The intensity of physical activity also plays a crucial role in oxygen delivery. High-intensity interval training (HIIT) induces greater spikes in CBF compared to moderate exercise, driven by increased metabolic stress and vascular demands (Sabag et al., 2021; Sudo et al., 2022; Jung et al., 2024). These effects not only enhance vascular responsiveness but also mitigate the recurrence of hypoxic pockets in critical brain areas, such as those observed during cortical spreading depression (Lu et al., 2021). Consistent aerobic exercise over time promotes angiogenesis, increases capillary density, and ensures sustained oxygen delivery, ultimately reducing the cumulative burden of hypoxia and supporting neuroplasticity (Sun et al., 2024). These mechanisms collectively decrease the risk of neurodegenerative conditions (Gorski and De Bock, 2019; Lamanna, 2018; Renke et al., 2022; Kantawala et al., 2023).

#### Social engagement impact

Social events engage neural circuits related to language, emotion, and executive function and increase regional blood flow and oxygenation. These interactions play a critical role in addressing hypoxic pockets by enhancing neurovascular coupling and directing oxygen delivery to under-perfused regions. Conversation, cooperation, and group activities require the stimulation of several different regions of the brain, especially the prefrontal and temporal cortices, where cognitive processing and emotional bonding occur (Rilling et al., 2008). Such interactions stimulate neuroplasticity and synaptogenesis, both of which contribute to cognitive resilience and adaptive responses to oxygen depletion. This increased activation in oxygen-demanding regions helps resolve transient oxygen deficits and reduces the recurrence of hypoxic episodes.

Additionally, social engagement provides protection against cognitive decline. Through enhanced neurovascular coupling, social engagement improves the delivery of oxygen to regions prone to hypoxia, such as the prefrontal cortex, thereby reducing the frequency of transient hypoxic episodes. Longitudinal studies have demonstrated that individuals with robust social networks experience slower cognitive deterioration and better recovery following neurological injury (Cammisuli et al., 2022; Xie et al., 2024; Levinger et al., 2023). Activities that combine social interactions with physical movements, such as group sports or dance, offer synergistic benefits by simultaneously enhancing neural and vascular health. These combined interventions are particularly effective in reducing localized oxygen deficits, resolving hypoxic pockets, and supporting long-term cognitive resilience (Levinger et al., 2023).

#### Cognitive stimulation

Cognitive challenges drive localized oxygen demand, prompting adaptive increases in blood flow to active brain regions. This heightened metabolic activity plays a critical role in resolving hypoxic pockets by directing oxygen delivery to areas of increased demand. Tasks involving learning, memory, and problem-solving increase metabolic demand, leading to increased neurovascular coupling and oxygen delivery (Robert et al., 2021; Zhu et al., 2022; Nishiguchi et al., 2015). For example, problem-solving tasks predominantly activate the prefrontal cortex, a region particularly susceptible to hypoxia, enhancing oxygenation to support executive functions and decision-making (Funahashi, 2022).

Engaging in sustained cognitive stimulation promotes both structural and functional changes in the brain, strengthened vascular networks, angiogenesis, and enhanced oxygen utilization efficiency, which reduce the persistence of hypoxic pockets in metabolically active regions like the hippocampus and prefrontal cortex (Beinlich et al., 2024; Wheeler et al., 2019). These mechanisms not only resolve transient hypoxia but also provide long-term protection against recurrent oxygen deficits. For instance, cognitive training exercises have been shown to increase capillary density and improve vascular responsiveness, ensuring that transient hypoxia is effectively managed during periods of high metabolic demand (Renke et al., 2022). These changes play crucial roles in helping the brain handle metabolic challenges and maintain mental sharpness as we age. Moreover, Wang et al. (2023) conducted a meta-analysis demonstrating that moderate-intensity exercise interventions positively impact executive functioning in children, indicating that the cognitive benefits of physical activity extend across different age groups. This synergy between cognitive stimulation and physical activity highlights the potential for multimodal interventions to address hypoxic pockets while simultaneously enhancing cognitive function.

### Clinical implications

Understanding how daily activities influence brain oxygenation offers important insights into both clinical practice and public health. The resolution of hypoxic pockets emerges as a promising target for interventions aimed at enhancing cognitive resilience and mitigating neurodegenerative decline. By utilizing physical, social, and cognitive activities as natural ways to regulate cerebral oxygen dynamics, interventions that foster cognitive resilience, slow neurodegenerative decline, and improve rehabilitation outcomes can be developed. This section focuses on three key areas: prevention, rehabilitation, and personalized intervention.

### Prevention strategies

Prevention strategies should focus on incorporating structured activities into public health initiatives to support cognitive health. Regular aerobic exercise, meaningful social interactions, and mental engagement activities have been shown to increase regional brain oxygenation and reduce the risk of cognitive decline and neurodegenerative diseases (Adjetey et al., 2023; Moriarty et al., 2019; Hayes et al., 2014; Liu et al., 2023). Specifically, these activities target hypoxic pockets by improving vascular responsiveness and neurovascular coupling, reducing both the frequency and severity of transient oxygen deficits. For example, walking groups or low-intensity sports tailored to older adults may directly improve capillary perfusion in vulnerable regions like the hippocampus and prefrontal cortex. These approaches are especially impactful when introduced early, particularly for individuals at greater risk of developing mild cognitive impairment (MCI).

In addition to structured activities, diversifying one’s daily routine—through pursuits like dancing, gardening, playing strategy games, or learning new skills—can further amplify neurovascular adaptations by engaging multiple brain regions (Joyce and Shaffer, 2016; Phillips, 2017). By promoting more robust neurovascular coupling and helping to resolve localized hypoxic pockets, such varied daily practices reinforce the protective effects of formal interventions on long-term cognitive resilience.

Accessible and cost-effective interventions such as community walking groups, educational workshops, and social clubs offer practical solutions for broad implementation. Additionally, public health campaigns emphasizing the connection between lifestyle behaviors and brain health can encourage the widespread adoption of preventative measures such as health campaigns can emphasize the connection between lifestyle choices and the mitigation of hypoxic pockets, encouraging individuals to adopt daily habits that support cerebral oxygenation.

#### Rehabilitation applications

Rehabilitation programs for cognitive impairment or neurological injuries can benefit from interventions that improve oxygen delivery to specific brain regions. For example, aerobic exercise has been shown to increase neurovascular coupling and stimulate angiogenesis in areas affected by stroke or traumatic brain injury (TBI), supporting recovery and enhancing cognitive outcomes (Cuccurullo et al., 2024; Toots et al., 2017; Yu et al., 2021). Pairing physical activity with cognitive training, such as practicing memory tasks while walking, can further amplify neuroplasticity and functional recovery. This combined approach addresses the intricate relationship between brain oxygenation, neural repair, and cognitive function, which leads to better long-term outcomes. Novel interventions such as partial-body cryostimulation have also been explored, showing potential benefits in terms of cognitive performance and cerebral oxygenation (Theurot et al., 2021). Pairing physical activity with cognitive training—such as practicing memory tasks while walking—can further amplify neuroplasticity and functional recovery. This combined approach addresses the intricate relationships among brain oxygenation, neural repair, and cognitive function, leading to better long-term outcomes.

#### Personalized approaches

The variability in brain oxygenation patterns highlights the need for personalized interventions that cater to individual differences. Advances in wearable sensors and imaging technologies, such as functional near-infrared spectroscopy (fNIRS), have enabled real-time monitoring of cerebral oxygenation. This capability allows clinicians to tailor activity programs by using objective biomarkers to ensure targeted and effective strategies (Herold et al., 2018; Bagyaraj et al., 2014). These tools can identify areas of chronic hypoxia and guide interventions to increase oxygen delivery and usage.

Personalized activity plans should also consider factors such as age, genetic background, and baseline cognitive abilities. For example, moderate exercise combined with social engagement may be more effective for older adults, whereas younger individuals might benefit from high-intensity cognitive tasks (Jackson et al., 2016; Lim et al., 2023; Nishiguchi et al., 2015). Using precision medicine principles to customize interventions not only maximizes their impact but also encourages long-term adherence to these beneficial activities.

### Future research directions

Despite accumulating evidence linking daily activities to enhanced brain perfusion and cognitive health, important knowledge gaps exist in terms of mechanistic pathways, optimal interventions, and long-term effects. Addressing these gaps requires further research that focuses on three key areas: mapping activity-specific effects, exploring individual variations, and advancing the development of targeted interventions.

#### Mapping activity-specific effects

Future studies would benefit greatly from employing advanced neuroimaging techniques to better understand how physical activities alter regional brain oxygenation and exert systemic effects on cognition. Portable near-infrared spectroscopy (fNIRS) offers valuable insights into how physical, social, and cognitive activities affect oxygen levels in specific areas of the human brain (Bagyaraj et al., 2014; Santos et al., 2019; Herold et al., 2018). Investigating the timing of these changes, whether they occur immediately or after a delay, is essential for identifying the most impactful life stages or events to target cognitive benefits.

Additionally, longitudinal studies are critical for evaluating how sustained engagement in these activities shapes brain structures and functions over time (Mulser and Moreau, 2023; Liu et al., 2023). These findings will inform the development of effective, enduring strategies for preserving cognitive health, guided by processes such as targeted neuronal growth, enhanced metabolic efficiency, and neuroplastic adaptations facilitated through consistent activity.

#### Individual variation studies

Research on individual responses to hypoxia provides valuable insights into adaptive mechanisms that enhance cognitive resilience. For instance, centenarians often demonstrate preserved cognitive abilities despite age-related physiological stressors, likely due to enhanced neurovascular remodeling and metabolic regulation (David et al., 2024). Similarly, studies of hypoxia-hypercapnia-tolerant mice reveal their ability to maintain normal activity levels under chronic low-oxygen conditions, offering models for understanding resilience to oxygen challenges (Tolstun et al., 2020).

Further, exploring the immediate and cumulative effects of physical and cognitive activities is crucial for refining intervention strategies. For example, does a single bout of exercise provide measurable oxygen delivery benefits, or are sustained adaptations required to achieve meaningful cognitive improvements? Understanding these temporal dynamics will help design interventions that balance short-term gains with long-term impacts (Sudo et al., 2022; Hayes et al., 2014).

Moreover, integrating nutritional interventions, such as those studied by Bloomfield et al. (2023), with physical and cognitive activities provides a holistic framework for resolving hypoxic pockets and optimizing brain oxygenation. Combining these strategies can enhance cognitive resilience, particularly in vulnerable populations like older adults.

#### Intervention development

Multimodal interventions that address the physical, cognitive, and social domains simultaneously show great potential for enhancing both brain oxygenation and cognitive health. However, these programs must be tailored to reflect the best available evidence of activity-specific effects and individual differences. For example, in a similar study, combining aerobic exercise with memory training affected a significantly broader range of molecular targets than did memory training alone, suggesting that aerobic exercise may synergize through processes such as improved neurovascular coupling and increased synaptic plasticity (Nishiguchi et al., 2015; Chen et al., 2024).

Implementation studies that address clinically embedded barriers to the uptake of these interventions include access, resource allocation, and participant engagement. Thus, the current utilization of wearable devices and mobile applications are prospective technologies that can support the long-term feedback of physiological indices underlying exercise performance (e.g., oxygenation) and adherence (Herold et al., 2018; Levinger et al., 2023). Moreover, these interventions should be implemented in conjunction with community-based organizations to allow scalability and sustainability for various population groups.

## Conclusion

Recent research underscores the profound impact of everyday activities on regulating brain oxygenation, revealing a practical and accessible pathway to address localized oxygen deficits. Physical exercise, social engagement, and cognitive stimulation emerge as powerful tools to offset these deficits over time, enhancing cognitive resilience and supporting overall brain function. These findings highlight the transformative potential of simple, modifiable behaviors in optimizing oxygen delivery to the brain, preventing cognitive decline, and aiding recovery from neurological injuries. By bridging lifestyle habits with region-specific brain oxygenation, this perspective offers a compelling framework for promoting cognitive health across diverse populations. However, critical questions remain about the biological mechanisms that underpin these effects and how they can be harnessed most effectively. Addressing these gaps will not only refine our understanding of brain oxygen dynamics but also enable the development of targeted, evidence-based interventions. These strategies have the potential to transform public health, rehabilitation, and personalized care, ultimately improving quality of life and fostering resilience across the lifespan.

## References

[B1] Acker T. Acker H. (2004). Cellular oxygen sensing need in CNS function: Physiological and pathological implications. *J. Exp. Biol.* 207(Pt 18), 3171–3188. 10.1242/jeb.01075 15299039

[B2] Adjetey C. Davis J. C. Falck R. S. Best J. R. Dao E. Bennett K. (2023). Economic evaluation of exercise or cognitive and social enrichment activities for improved cognition after stroke. *JAMA Netw. Open* 6:e2345687. 10.1001/jamanetworkopen.2023.45687 PMC10690466 38032638

[B3] Arefirad T. Seif E. Sepidarkish M. Mohammadian Khonsari N. Mousavifar S. A. Yazdani S. (2022). Effect of exercise training on nitric oxide and nitrate/nitrite (NOx) production: A systematic review and meta-analysis. *Front. Physiol.* 13:953912. 10.3389/fphys.2022.953912 36267589 PMC9576949

[B4] Bagyaraj S. Devi S. S. Ravindran G. (2014). Design and development of two channel portable near infrared spectroscopy system for studies of cerebral oxygenation in prefrontal cortex of human brain. *Appl. Mech. Mater.* 573 814–818. 10.4028/www.scientific.net/amm.573.814

[B5] Beinlich F. R. M. Asiminas A. Untiet V. Bojarowska Z. Plá V. Sigurdsson B. (2024). Oxygen imaging of hypoxic pockets in the mouse cerebral cortex. *Science* 383 1471–1478. 10.1126/science.adn1011 38547288 PMC11251491

[B6] Benarroch E. (2022). What muscle signals mediate the beneficial effects of exercise on cognition? *Neurology* 99 298–304. 10.1212/wnl.0000000000201049 35970575

[B7] Bloomfield P. M. Fisher J. P. Shaw D. M. Gant N. (2023). Cocoa flavanols protect cognitive function, cerebral oxygenation, and mental fatigue during severe hypoxia. *J. Appl. Physiol.* 135 475–484. 10.1152/japplphysiol.00219.2023 37471213

[B8] Bon L. I. Fliuryk S. Dremza I. Bon E. Maksimovich N. Prykhodzka A. (2023). Hypoxia of the brain and mechanisms of its development. *J. Clin. Res. Rep.* 13:311. 10.31579/2690-1919/311

[B9] Cammisuli D. M. Castelnuovo G. Fusi J. Scarfò G. Franzoni F. Martini C. (2022). What does the brain have to keep working at its best? resilience mechanisms such as antioxidants and brain/cognitive reserves for counteracting Alzheimer’s disease degeneration. *Biology* 11:650. 10.3390/biology11050650 35625381 PMC9138251

[B10] Chen W. Siew-Pin J. L. Wu Y. Huang N. Teo W. (2024). Identifying exercise and cognitive intervention parameters to optimize executive function in older adults with mild cognitive impairment and dementia: A systematic review and meta-analyses of randomized controlled trials. *Eur. Rev. Aging Phys. Activity* 21:22. 10.1186/s11556-024-00357-4 39215230 PMC11363393

[B11] Cuccurullo S. J. Fleming T. K. Hanley D. F. Raghavan P. Petrosyan H. (2024). Mechanisms and benefits of cardiac rehabilitation in individuals with stroke: Emerging role of its impact on improving cardiovascular and neurovascular health. *Front. Cardiovasc. Med.* 11:1376616. 10.3389/fcvm.2024.1376616 38756753 PMC11096558

[B12] David E. Wolfson M. Muradian K. K. Fraifeld V. E. (2024). The potential longevity-promoting hypoxic-hypercapnic environment as a measure for radioprotection. *Biogerontology* 25, 891–898. 10.1007/s10522-024-10129-3 39162980 PMC11374852

[B13] Friedrich J. Janssen F. Aleynik D. Bange H. W. Boltacheva N. Çağatay M. N. (2013). Investigating hypoxia in aquatic environments: Diverse approaches to addressing a complex phenomenon. *Biogeosciences* 11 1215–1259. 10.5194/bg-11-1215-2014

[B14] Funahashi S. (2022). *Dorsolateral Prefrontal Cortex.* Berlin: Springer Nature Singapore, 1–51.

[B15] Gorski T. De Bock K. (2019). Metabolic regulation of exercise-induced angiogenesis. *Vascular Biol.* 1 H1–H8. 10.1530/vb-19-0008 32923947 PMC7439921

[B16] Hakun J. G. Benson L. Qiu T. Elbich D. B. Katz M. Shaw P. A. (2024). Cognitive health benefits of everyday physical activity in a diverse sample of middle-aged adults. *Ann. Behav. Med. A Publ. Soc. Behav. Med.* 10.1093/abm/kaae059 Online ahead of print. 39427230 PMC11783295

[B17] Hayes S. M. Alosco M. L. Forman D. E. (2014). The effects of aerobic exercise on cognitive and neural decline in aging and cardiovascular disease. *Curr. Geriatrics Rep.* 3 282–290. 10.1007/s13670-014-0101-x 25750853 PMC4349343

[B18] Herold F. Wiegel P. Scholkmann F. Müller N. G. (2018). Applications of functional near-infrared spectroscopy (fNIRS) neuroimaging in exercise-cognition science: A systematic, methodology-focused review. *J. Clin. Med.* 7:466. 10.3390/jcm7120466 30469482 PMC6306799

[B19] Hoffmann A. Kunze R. Helluy X. Milford D. Heiland S. Bendszus M. (2016). High-field MRI reveals a drastic increase of hypoxia-induced microhemorrhages upon tissue reoxygenation in the mouse brain with strong predominance in the olfactory bulb. *PLoS One* 11:e0148441. 10.1371/journal.pone.0148441 26863147 PMC4749302

[B20] Hohenauer E. Freitag L. Costello J. T. Williams T. B. Küng T. Taube W. (2022). The effects of normobaric and hypobaric hypoxia on cognitive performance and physiological responses: A crossover study. *PLoS One* 17:e0277364. 10.1371/journal.pone.0277364 36355846 PMC9648783

[B21] Jackson P. A. Pialoux V. Corbett D. Drogos L. Erickson K. I. Eskes G. A. (2016). Promoting brain health through exercise and diet in older adults: A physiological perspective. *J. Physiol.* 594 4485–4498. 10.1113/JP271270 27524792 PMC4983622

[B22] Joyce K. E. Shaffer H. J. (2016). Neuroplasticity and clinical practice: Building brain power for health. *Front. Psychol.* 7:1118. 10.3389/fpsyg.2016.01118 27507957 PMC4960264

[B23] Jung M. Pontifex M. B. Hillman C. H. Kang M. Voss M. W. Erickson K. I. (2024). A mechanistic understanding of cognitive performance deficits concurrent with vigorous intensity exercise. *Brain Cogn.* 180:106208. 10.1016/j.bandc.2024.106208 39111187

[B24] Kantawala B. Ramadan N. Hassan Y. Fawaz V. Mugisha N. Nazir A. (2023). Physical activity intervention for the prevention of neurological diseases. *Health Sci. Rep.* 6:e1524. 10.1002/hsr2.1524 37614284 PMC10442603

[B25] Lamanna J. C. (2018). Cerebral angioplasticity: The anatomical contribution to ensuring appropriate oxygen transport to brain. *Adv. Exp. Med. Biol.* 1072 3–6. 10.1007/978-3-319-91287-5_1 30178315

[B26] Levinger P. Goh A. M. Y. Dunn J. Katite J. Paudel R. Onofrio A. (2023). Exercise intervention outdoor project in the community—results from the ENJOY program for independence in dementia: A feasibility pilot randomized controlled trial. *BMC Geriatrics* 23:426. 10.1186/s12877-023-04132-5 37438710 PMC10337151

[B27] Li M.-Y. Huang M.-M. Li S.-Z. Tao J. Zheng G.-H. Chen L.-D. (2016). The effects of aerobic exercise on the structure and function of DMN-related brain regions: A systematic review. *Int. J. Neurosci.* 127 634–649. 10.1080/00207454.2016.1212855 27412353

[B28] Lim S. Ibrahim K. Clift E. Meredith S. Roberts H. C. Agnew S. (2023). 1323 volunteer-led online group exercise for older adults: A feasibility and acceptability study. *Age Aging* 52 (Suppl. 1), 10.1093/ageing/afac322.047 PMC10375749 37507667

[B29] Liu X. Ma Z. Zhu X. Zheng Z. Li J. Fu J. (2023). Cognitive benefit of a multidomain intervention for older adults at risk of cognitive decline: A cluster-randomized controlled trial. *Am. J. Geriatric Psychiatry* 31 197–209. 10.1016/j.jagp.2022.10.006 36414488

[B30] Lu Y. Zhou X. Cheng J. Ma Q. (2021). Early intensified rehabilitation training with hyperbaric oxygen therapy improves functional disorders and prognosis of patients with traumatic brain injury. *Adv. Wound Care* 10 663–670. 10.1089/wound.2018.0876 34546088 PMC8568788

[B31] Moriarty T. A. Beltz N. Kravitz L. Gibson A. Mermier C. Zuhl M. (2019). Acute aerobic exercise based cognitive and motor priming: Practical applications and mechanisms. *Front. Psychol.* 10:02790. 10.3389/fpsyg.2019.02790 31920835 PMC6920172

[B32] Mulser L. Moreau D. (2023). Effect of acute cardiovascular exercise on cerebral blood flow: A systematic review. *Brain Res.* 1809:148355. 10.1016/j.brainres.2023.148355 37003561

[B33] Murayama Y. Sato Y. Hu L. Sakatani K. Compare A. Brugnera A. (2017). Relation between cognitive function and baseline concentrations of hemoglobin in prefrontal cortex of elderly people measured by time-resolved near-infrared spectroscopy. *Adv. Exp. Med. Biol.* 977 269–276. 10.1007/978-3-319-55231-6_37 28685456

[B34] Nishiguchi S. Yamada M. Tanigawa T. Sekiyama K. Kawagoe T. Suzuki M. (2015). A 12-week physical and cognitive exercise program can improve cognitive function and neural efficiency in community-dwelling older adults: A randomized controlled trial. *J. Am. Geriatrics Soc.* 63 1355–1363. 10.1111/jgs.13481 26114906

[B35] Phillips C. (2017). Lifestyle modulators of neuroplasticity: How physical activity, mental engagement, and diet promote cognitive health during aging. *Neural Plasticity* 2017:3589271. 10.1155/2017/3589271 28695017 PMC5485368

[B36] Popa-Wagner A. Buga A. M. Popescu B. Muresanu D. (2015). Vascular cognitive impairment, dementia, aging and energy demand. A vicious cycle. *J. Neural Transmission* 122 (Suppl. 1), S47–S54. 10.1007/s00702-013-1129-3 24337666

[B37] Popa-Wagner A. Buga A. M. Tica A. A. Albu C. V. (2014). Perfusion deficits, inflammation and aging precipitate depressive behaviour. *Biogerontology* 15 439–448. 10.1007/s10522-014-9516-1 25033986

[B38] Qiao M. Malisza K. Del Bigio M. Tuor U. (2002). Transient hypoxia-ischemia in rats: Changes in diffusion-sensitive MR imaging findings, extracellular space, and Na+-K+-adenosine triphosphatase and cytochrome oxidase activity. *Radiology* 223 65–75. 10.1148/radiol.2231010736 11930049

[B39] Renke M. B. Marcinkowska A. B. Kujach S. Winklewski P. J. (2022). A systematic review of the impact of physical exercise-induced increased resting cerebral blood flow on cognitive functions. *Front. Aging Neurosci.* 14:803332. 10.3389/fnagi.2022.803332 35237146 PMC8882971

[B40] Rilling J. K. Dagenais J. E. Goldsmith D. R. Glenn A. L. Pagnoni G. (2008). Social cognitive neural networks during in-group and out-group interactions. *NeuroImage* 41 1447–1461. 10.1016/j.neuroimage.2008.03.044 18486491

[B41] Robert J. Caffrey T. Button E. (2021). Toward three-dimensional in vitro models to study neurovascular unit functions in health and disease. *Neural Regeneration Res.* 16:2132. 10.4103/1673-5374.310671 33818484 PMC8354124

[B42] Sabag A. Johnson N. A. Little J. P. (2021). Low-volume high-intensity interval training for cardiometabolic health. *J. Physiol.* 600 1013–1026. 10.1113/jp281210 33760255

[B43] Salzman T. Dupuy O. Fraser S. A. (2022). Effects of cardiorespiratory fitness on cerebral oxygenation in healthy adults: A systematic review. *Front. Physiol.* 13:838450. 10.3389/fphys.2022.838450 35309063 PMC8931490

[B44] Santos E. A. B. Rodrigues M. A. B. Lucena R. J. R. S. Lins L. T. Lima E. G. (2019). *Low-Cost Functional Near Infrared Spectroscopy (fNIRS) Applied on Brain-Computer Interfaces (BCIs).* Berlin: Springer Singapore, 495–500.

[B45] Stoica S. Bleotu C. Ciobanu V. Ionescu A. Albadi I. Onose G. (2022). Considerations about hypoxic changes in neuraxis tissue injuries and recovery. *Biomedicines* 10:381. 10.3390/biomedicines10020481 35203690 PMC8962344

[B46] Sudo M. Costello J. T. McMorris T. Ando S. (2022). The effects of acute high-intensity aerobic exercise on cognitive performance: A structured narrative review. *Front. Behav. Neurosci.* 16:957677. 10.3389/fnbeh.2022.957677 36212191 PMC9538359

[B47] Sun H. Zhang Y. Shi L. (2024). Advances in exercise-induced vascular adaptation: Mechanisms, models, and methods. *Front. Bioeng. Biotechnol.* 12:1370234. 10.3389/fbioe.2024.1370234 38456010 PMC10917942

[B48] Takano T. Tian G.-F. Peng W. Lou N. Lovatt D. Hansen A. J. (2007). Cortical spreading depression causes and coincides with tissue hypoxia. *Nat. Neurosci*. 10 754–762. 10.1038/NN1902 17468748

[B49] Theurot D. Dugué B. Douzi W. Guitet P. Louis J. Dupuy O. (2021). Impact of acute partial-body cryostimulation on cognitive performance, cerebral oxygenation, and cardiac autonomic activity. *Sci. Rep.* 11:7793. 10.1038/s41598-021-87089-y 33833278 PMC8032750

[B50] Tolstun D. A. Knyazer A. Tushynska T. V. Dubiley T. A. Bezrukov V. V. Fraifeld V. E. (2020). Metabolic remodeling of mice by hypoxic-hypercapnic environment: Imitating the naked mole-rat. *Biogerontology* 21 143–153. 10.1007/s10522-019-09848-9 31667660

[B51] Toots A. Littbrand H. Boström G. Hörnsten C. Holmberg H. Lundin-Olsson L. (2017). Effects of exercise on cognitive function in older people with dementia: A randomized controlled trial. *J. Alzheimer’s Dis.* 60 323–332. 10.3233/JAD-170014 28800328 PMC5611799

[B52] Veenith T. V. Carter E. L. Geeraerts T. Grossac J. Newcombe V. F. Outtrim J. (2016). Pathophysiologic mechanisms of cerebral ischemia and diffusion hypoxia in traumatic brain injury. *JAMA Neurol.* 73 542–550. 10.1001/jamaneurol.2016.0091 27019039

[B53] Wang H. Yang Y. Xu J. Niu L. Zhang Y. Shi J. (2023). Meta-analysis on the effects of moderate-intensity exercise intervention on executive functioning in children. *PLoS One* 18:e0279846. 10.1371/journal.pone.0279846 36812181 PMC9946206

[B54] Wheeler M. J. Dunstan D. W. Smith B. Smith K. J. Scheer A. Lewis J. (2019). Morning exercise mitigates the impact of prolonged sitting on cerebral blood flow in older adults. *J. Appl. Physiol.* 126 1049–1055. 10.1152/japplphysiol.00001.2019 30730813 PMC6485691

[B55] Willie C. K. Cowan E. C. Ainslie P. N. Taylor C. E. Smith K. J. Sin P. Y. W. (2011). Neurovascular coupling and distribution of cerebral blood flow during exercise. *J. Neurosci. Methods* 198 270–273. 10.1016/j.jneumeth.2011.03.017 21459113

[B56] Xie X. Xu A. Lyu Y. Wu F. Zong A. Zhuang Z. (2024). Exploring the association between multidimensional social isolation and heterogeneous cognitive trajectories among older adults: Evidence from China. *Front. Public Health* 12:1426723. 10.3389/fpubh.2024.1426723 39421814 PMC11484626

[B57] Xue Y. Georgakopoulou T. van der Wijk A. E. Józsa T. I. van Bavel E. Payne S. J. (2022). Quantification of hypoxic regions distant from occlusions in cerebral penetrating arteriole trees. *PLoS Computat. Biol.* 18:e1010166. 10.1371/journal.pcbi.1010166 35930591 PMC9385041

[B58] Yu F. Vock D. M. Zhang L. Salisbury D. Nelson N. W. Chow L. S. (2021). Cognitive effects of aerobic exercise in Alzheimer’s disease: A pilot randomized controlled trial. *J. Alzheimer’s Dis.* 80 233–244. 10.3233/JAD-201100 33523004 PMC8075384

[B59] Zhang L. Y. Hu Y. Y. Liu X. Y. Wang X. Y. Li S. C. Zhang J. G. (2024). The role of astrocytic mitochondria in the pathogenesis of brain ischemia. *Mol. Neurobiol.* 61 2270–2282. 10.1007/s12035-023-03714-z 37870679

[B60] Zhou H. Wang J. Zhu Z. Hu L. An E. Lu J. (2024). A new perspective on stroke research: Unraveling the role of brain oxygen dynamics in stroke pathophysiology. *Aging Dis.* 10.14336/ad.2024.0548 Online ahead of print. 39226161 PMC12221400

[B61] Zhu W. M. Neuhaus A. Sutherland B. A. Beard D. J. Deluca G. C. (2022). Neurovascular coupling mechanisms in health and neurovascular uncoupling in Alzheimer’s disease. *Brain* 145 2276–2292. 10.1093/brain/awac174 35551356 PMC9337814

